# A Remotely Delivered Exercise-Based Rehabilitation Program for Patients with Persistent Chemotherapy-Induced Peripheral Neuropathy (EX-CIPN): A Phase I Feasibility Trial

**DOI:** 10.3390/curroncol33100621

**Published:** 2026-10-15

**Authors:** Eric M. Antonen, Michelle B. Nadler, David M. Langelier, Kristin L. Campbell, David Flamer, Jang Hyuk Cho, Scott Capozza, Lisa Avery, Kelcey A. Bland, Scott Leatherdale, Jackie Manthorne, Muna Al-Khaifi, Jennifer M. Jones

**Affiliations:** 1 Department of Supportive Care, Princess Margaret Cancer Centre, Toronto, ON M5G 2C4, Canada; 2 Institute of Medical Sciences, University of Toronto, Toronto, ON M5S 3K9, Canadadavid.langelier@cancercarealberta.ca (D.M.L.); 3 Division of Medical Oncology & Hematology, Princess Margaret Cancer Centre, Toronto, ON M5G 2C4, Canada; 4 Department of Oncology, Cumming School of Medicine, University of Calgary, Calgary, AB T2N 2T8, Canada; 5 Department of Physical Therapy, University of British Columbia, Vancouver, BC V6T 1Z3, Canada; 6 Department of Anesthesiology & Pain Medicine, University of Toronto, Toronto, ON M5G 1E2, Canada; 7 Department of Rehabilitation Medicine, Keimyung University Dongsan Hospital, Dongsan 42601, Republic of Korea; 8 Rehabilitation Department, Yale New Haven Hospital, New Haven, CT 06511, USA; 9 School of Public Health Sciences, University of Waterloo, Waterloo, ON N2L 3G5, Canada; 10 Canadian Cancer Survivor Network, Ottawa, ON K2C 2B5, Canada; 11 Department of Surgery, Mount Sinai Hospital, Toronto, ON M6G 1X5, Canada

**Keywords:** chemotherapy-induced peripheral neuropathy, survivorship, rehabilitation, exercise oncology, mixed-methods

## Abstract

Background: Chemotherapy-induced peripheral neuropathy (CIPN) affects two-thirds of patients who receive neurotoxic chemotherapy. In almost half of these patients, CIPN is persistent, with limited treatment options. Exercise-based rehabilitation shows promising results in improving CIPN-related outcomes, but high-quality research is needed with CIPN symptoms and disability as primary endpoints. Methods: We conducted a phase I single-arm, multi-method pilot study of a 10-week remotely delivered exercise-based rehabilitation program (EX-CIPN) for adult (≥18-years) cancer survivors experiencing CIPN symptoms ≥6 months post-chemotherapy. Primary outcomes were feasibility (accrual, retention, and adherence), acceptability (interviews and surveys), and safety (CTCAE v5). Secondary outcomes included pain, CIPN symptoms, CIPN-related disability, and physiologic assessments. Assessments were conducted at baseline (T1), post-program (T2), and 3 months post-program (T3). Post-program feedback surveys were completed at T2 and semi-structured qualitative video interviews were completed between T2 and T3. Results: In total, 36 participants (median age: 59, 83% female) consented to participate in the study. T1 retention rate (90%), adherence rates (77–96%), and no reported serious adverse events (Grade 3 CTCAE v5) supported study feasibility and safety. Interviews and surveys supported intervention acceptability. Retention rates at T2 (68%) and T3 (63%) did not reach feasibility targets. CIPN symptoms and CIPN-related disability showed significant improvement at T3 (*p* < 0.001). Conclusions: Results suggest that EX-CIPN has encouraging feasibility outcomes and is acceptable and safe. Preliminary clinical outcome results are promising. Based on these study results, we can proceed to test this intervention, with a few modifications, in a prospective randomised trial with a CIPN-related primary outcome.

## Simple Summary

Two-thirds of cancer patients who receive neurotoxic chemotherapy will experience chemotherapy-induced peripheral neuropathy (CIPN). Symptoms of CIPN can continue for months or years after chemotherapy and can impact patients’ balance, strength, and ability to complete everyday tasks. Currently, there are very few options to treat CIPN symptoms. Exercise has shown the potential to help alleviate symptoms of CIPN; however, more research is needed before exercise can be prescribed regularly to patients with CIPN. Our research aims to determine if a remote exercise-based rehabilitation program (EX-CIPN) designed to address CIPN symptoms and related issues with physical function is feasible for a CIPN population. We found that the EX-CIPN program was feasible, acceptable, and safe for patients experiencing CIPN. We were also able to show promising preliminary findings of the program’s effects on CIPN symptoms and CIPN-related disability. Larger scale, randomised testing of this program is needed to confirm this effectiveness.

## 1. Introduction

Early detection and improvements in cancer treatments have led to an increase in the number of people living with a history of cancer [1]. Over 2 million Canadians report being diagnosed with cancer during their lifetime [2] and this number is expected to increase over the next decade. As individuals transition into survivorship, the long-term effects of cancer treatments, morbidity, and related disability are of increasing importance to improve and maintain quality of life (QoL) [3].

Chemotherapy-induced peripheral neurotoxicity (CIPN) is a common, debilitating side effect that develops in approximately 68% of patients who receive neurotoxic treatments, such as taxane and platinum-based chemotherapies [4,5]. While acute symptoms can improve spontaneously within 3 to 6 months after treatment [4], up to 40% of patients will experience persistent CIPN lasting months, years, or indefinitely [6,7].

CIPN presents primarily as a sensory neuropathy affecting the distal limbs and may cause numbness, tingling, burning sensations, pain, temperature sensitivity, and loss of proprioception [5]. These impairments can contribute to gait disturbances, reduced balance, muscle weakness due to disuse, and physical deconditioning, all which increase fall risk [8,9]. CIPN can also negatively impact patients’ QoL. Colon and lung cancer patients experiencing CIPN report worsened global QoL as well as physical, role, emotional, cognitive, and social functioning [10,11]. Persistent CIPN, defined as symptoms lasting longer than 6 months after chemotherapy completion [12], has limited symptomatic treatment options. Pharmacological treatments that exist are often associated with side effects [13,14]. Due to the uncertain effectiveness and side effects of pharmacologic treatments, many cancer survivors prefer to avoid additional medications [15]. Exercise-based interventions are a non-pharmacologic option for the symptomatic treatment of CIPN and have shown encouraging positive effects on CIPN-related outcomes [13,16,17,18]. These positive effects have been seen across different exercise types prescribed at different timepoints during treatment. A pilot randomised trial of a hand-specific exercise program in patients currently receiving chemotherapy found that decline in activities of daily living and pain was significantly improved in the intervention compared to control [19]. It was also shown, in patients during and after chemotherapy, that a program combining hand and foot sensory and strengthening exercises was able to improve neuropathy symptoms and quality of life [20]. Despite this promising evidence, the current literature is limited by low study quality and a lack of adequately powered, rigorous trials evaluating CIPN symptoms and disability as primary endpoints [13,18]. Supervised virtual exercise interventions have been shown to be feasible, acceptable, and safe and cancer survivors report a preference for exercising at home if provided with the right support [21,22,23,24,25]. Finally, the incorporation of behaviour change theory and techniques is recommended in interventions that require the adoption of health behaviours such as exercise [26]. Despite this, there are no studies evaluating exercise based interventions for persistent CIPN that incorporate behaviour change theories/frameworks or that are remotely delivered [27].

To address existing knowledge gaps, we developed a 10-week remote, exercise-based rehabilitation program (EX-CIPN) for cancer survivors experiencing persistent CIPN that incorporates behaviour change theory and techniques. The purpose of this study was to evaluate the feasibility, acceptability, and safety of the EX-CIPN program and to provide preliminary evidence on effectiveness. If the intervention is feasible, estimates of effectiveness would allow the development of an appropriately powered randomised trial with a CIPN-related primary outcome.

## 2. Methods

### 2.1. Study Design

This was a Phase I single-center, multi-method, single-arm, pilot feasibility study of the EX-CIPN intervention with patients experiencing persistent CIPN at Princess Margaret Cancer Centre, Toronto, Canada. Following screening and consent, study participants completed 3 assessment time-points throughout the study made up of a questionnaire package and in-person physical assessment. These assessments included: a baseline assessment (T1), a follow-up assessment immediately post-intervention at 10 weeks (T2), and a final follow-up assessment 3 months post-intervention at 22 weeks (T3). The trial was registered with clinicaltrials.gov (NCT06405542) and was approved by the University Health Network Research Ethics Board (REB# 23-5839). For the full study protocol refer to the study protocol paper [28].

### 2.2. Study Population

Participants were eligible if they were: (a) ≥18 years of age; (b) diagnosed with any type of cancer; (c) received chemotherapy treatment with curative intent (no minimum dose); (d) ≥6 months following chemotherapy completion; (e) reported ≥Grade 1 on the numbness and tingling severity item of the Common Terminology Criteria for Adverse Events (CTCAE) v5.0 [29], meaning mild to severe symptoms and neuropathic pain ≥ 3 on the Neuropathic Pain 4 (DN4 interview) (0–7) [30]; (f) engaged in <90 min per week of moderate-intensity aerobic exercise; (g) independent with ambulation and transfers with or without ambulatory assistance; (h) able to communicate sufficiently in English to complete intervention, questionnaires, and consent; and (i) had access to and were able to operate videoconferencing. Patients receiving maintenance oncologic therapies not associated with neuropathy (e.g., endocrine therapy or PARP inhibitors) were included. They were also permitted to continue oral, topical, or regional medications/interventions for CIPN at study entry, provided that the dose and treatment approach had remained stable for at least six weeks prior to enrollment and neuropathy symptoms persisted. Patients were excluded if they had neurological conditions that limited their ability to safely or appropriately participate in the prescribed exercise program, if they had pre-existing neuropathy prior to chemotherapy, or if they were participating in another rehabilitation or exercise-based intervention.

During the study period, participants were instructed not to modify the dosage of neuropathy-related medications, creams, or complementary therapies, nor to initiate any new treatments for neuropathy. Adherence to these requirements was monitored by the study team and documented at all assessment timepoints, T1 through T3, as well as during each health coaching call.

### 2.3. Intervention

The EX-CIPN program is a 10-week remote exercise-based rehabilitation program prescribed and supported by a Registered Kinesiologist (RKin). Participants met with the RKin via videoconference technology (MS Teams^®^ version 25195.1002.3963.7410, Redmond, WA, USA) to develop and progress their individualised, remote exercise program. Participants received five videoconference appointments spaced out over the 10 weeks to modify or progress their program as needed, review exercise adherence and goals, and identify any potential exercise barriers. The progressive exercise program included aerobic, resistance, and flexibility training, along with CIPN-specific exercises, based on previous literature on exercise in CIPN [17,19,20,31,32,33]. CIPN-specific exercises targeted the hands and the feet with potential exercise types including strengthening, desensitisation, and nerve flossing. Previous evidence on established behaviour change theory and techniques was also used to promote exercise uptake [34,35,36,37,38,39]. Different theoretical models were built into the intervention including cognitive behavioural therapy and transtheoretical model of behaviour change [34,36]. The RKins delivering the intervention were trained in motivational interviewing through the evaluation and encouragement of intrinsic motivation, the strengthening of self-efficacy, and the use of a collaborative problem-solving approach [39]. This can strengthen participants’ motivation for change and has been shown to be effective in populations with cancer and other chronic conditions [39,40]. The program is also supported by a mobile application (Physitrack^®^ version 5.8.1, London, UK) that allows participants to view exercise video and descriptions from home. Participants were provided wearable technology in the form of a Fitbit™, Google LLC, San Francisco, CA, USA for remote monitoring. This allowed the prescribing RKin to track activity and promoted participant behaviour change through self-monitoring [41]. Behaviour change techniques used in EX-CIPN can be seen in Table 1.

### 2.4. Study Outcomes and Data Collection

#### 2.4.1. Demographics

Demographic data were collected at baseline including: age, gender, ethnicity, educational background, employment status, socioeconomic status, living arrangement, initial cancer diagnosis, cancer stage, type of chemotherapy, and time of initial diagnosis.

#### 2.4.2. Feasibility

Feasibility was evaluated based on accrual, retention, and adherence to the intervention. Consistent with the Consolidated Standards of Reporting Trials guidelines for pilot and feasibility studies [42], predefined targets for accrual, retention, and adherence were established a priori using benchmarks from relevant literature [43,44]. The target for accrual was 4 participants per month, the target for retention was ≥70% at each study timepoint, and the target for adherence was ≥70% adherence to each intervention component.

Retention was defined as the percentage of participants who completed assessments at T1, T2, and T3. Rates of complete and missing data were also collected.

The intervention components used to measure adherence were check-in calls with the RKin and self-report completion of the weekly exercise plan (aerobic, resistance and flexibility). Adherence to check-in calls was measured by the percentage of check-in calls completed versus prescribed. Adherence to aerobic exercise was measured by the number of minutes per week completed divided by the number prescribed and adherence to resistance and flexibility programming was measured by the number of days per week that the program was completed divided by the number of days prescribed.

#### 2.4.3. Acceptability

Acceptability of the EX-CIPN intervention was measured using individual semi-structured qualitative video interviews and post-program feedback surveys. Post-program feedback surveys were completed at the T2 assessment and qualitative interviews were completed between the T2 and T3 assessments. Interview participants were selected sequentially through convenience sampling. Out of 14 participants approached, 8 completed an interview.

#### 2.4.4. Safety

All adverse events were graded according to CTCAE version 5.0 [29] and recorded during scheduled check-in visits and follow-up assessments. Safety events were clarified with study team physicians to ensure accurate association and grading.

#### 2.4.5. Secondary Outcomes

Secondary outcomes included the following patient-reported outcome measures (PROMs): (1) numeric pain rating scale (NPRS) measuring pain [45], (2) European Organisation for Research and Treatment of Cancer Quality of Life Questionnaire—Chemotherapy-Induced Peripheral Neuropathy 20-item scale (EORTC CIPN-20) measuring CIPN symptoms [46], and (3), Rasch-built Overall Disability Scale for patients with chemotherapy-induced peripheral neuropathy (CIPN-R-ODS) measuring CIPN-related disability [47]. The NPRS is a widely used, valid, and reliable measure asking patients to rate their pain on a numerical scale (0–10) [48]. The EORTC CIPN-20 is a validated 20-item questionnaire assessing CIPN symptoms and severity [49]. The CIPN-R-ODS is a 28-item measure evaluating the impact that CIPN has on daily tasks and activities [47]. Physiologic assessments used included: (1) grip dynamometer (bilateral hand-grip strength) [50], (2) 30 s sit-to-stand (lower-extremity strength) [51], (3) 7-item Berg Balance Test (balance) [52], (4) 4 metre gait speed (gait speed) [53], and (5) 6 min walk test (6MWT) (exercise capacity) [54].

### 2.5. Data Analysis

#### 2.5.1. Sample Size

There is no definitive recommended sample size or established guideline for determining the sample size of Phase I pilot studies assessing feasibility and acceptability. Therefore, simulations were conducted to examine the precision of estimates across a range of sample sizes and standard deviations (α = 0.05, power = 80%). These simulations indicated that a sample size of approximately 35–40 represented the elbow point of the resulting curves, suggesting diminishing gains in precision beyond this range. Accordingly, a sample size of 40 was selected and considered sufficient to assess feasibility within the proposed study design [55,56]. This sample size is also consistent with targets used in similarly designed studies evaluating feasibility and acceptability [57,58].

#### 2.5.2. Primary Outcomes

Study feasibility was evaluated using descriptive statistics of accrual rate, participant consent, and retention rates at all study time-points. Intervention feasibility was evaluated using descriptive statistics of adherence rates to all intervention components.

Acceptability was evaluated using descriptive statistics from post-program feedback surveys combined with thematic analysis of qualitative interviews. Thematic analysis of the transcripts was conducted using Braun and Clarke’s six-phase framework [59]. These phases include: (1) Data familiarisation, (2) initial code generation, (3) constructing themes, (4) reviewing themes, (5) revising and defining themes, and (6) producing the report [59]. The initial generation of codes was performed deductively with pre-established categories developed to reflect the core elements of the program and to systematically capture participants’ experiences, including perceived benefits, limitations, and suggested areas for improvement. The interviews were analysed with these first, however, while going through the transcripts, reviewers developed additional codes inductively and added these to the codebook. Themes were generated by identifying relationships among the codes. The reviewers then assessed each theme to ensure it was supported by sufficient data and demonstrated internal consistency. Following this review, the two reviewers refined the themes by combining similar codes and removing codes that lacked sufficient supporting data. The qualitative data from the codebook was then used to help explain post-program feedback surveys.

All program-related safety events were recorded in accordance with the Common Terminology Criteria for Adverse Events version 5.0 [60]. Each event was categorized based on its assessed relationship to the intervention (definitely related, possibly related, or unrelated).

#### 2.5.3. Secondary Outcomes

To support future sample size planning, completion rates for PROMs were evaluated and reported at each assessment point. The study was not powered to detect within-person changes from baseline to follow-up across T1 to T3. Instead, these analyses were conducted to generate preliminary estimates of effect sizes and corresponding 95 percent confidence intervals. Hedge’s g standardised mean difference effect sizes were calculated for the change from T1 to T2 and T1 to T3. Baseline distributions for all outcome measures were also summarised. Exploratory mixed-effects models were applied to estimate changes between T1 and T2 and between T2 and T3, using all available patient data under a missing at random assumption.

#### 2.5.4. Missing Data

All observed data was included in the mixed effects model, participants only observed at one or two timepoints still contributed to the model estimates. No data was imputed.

## 3. Results

### 3.1. Participant Baseline Information

A total of 40 participants consented to the study and 36 participants provided baseline demographics. The participant demographic data are presented in Table 2. The mean age of participants was 60.3 years, with most participants identifying as female (*n* = 30, 83%) and Caucasian (*n* = 25, 69%). The most common cancer diagnoses among the sample were breast cancer (*n* = 16, 44%) and ovarian cancer (*n* = 11, 31%). Three participants were diagnosed with stage IV cancer; however, all were treated curatively.

### 3.2. Primary Outcomes—Feasibility

**Figure 1:**
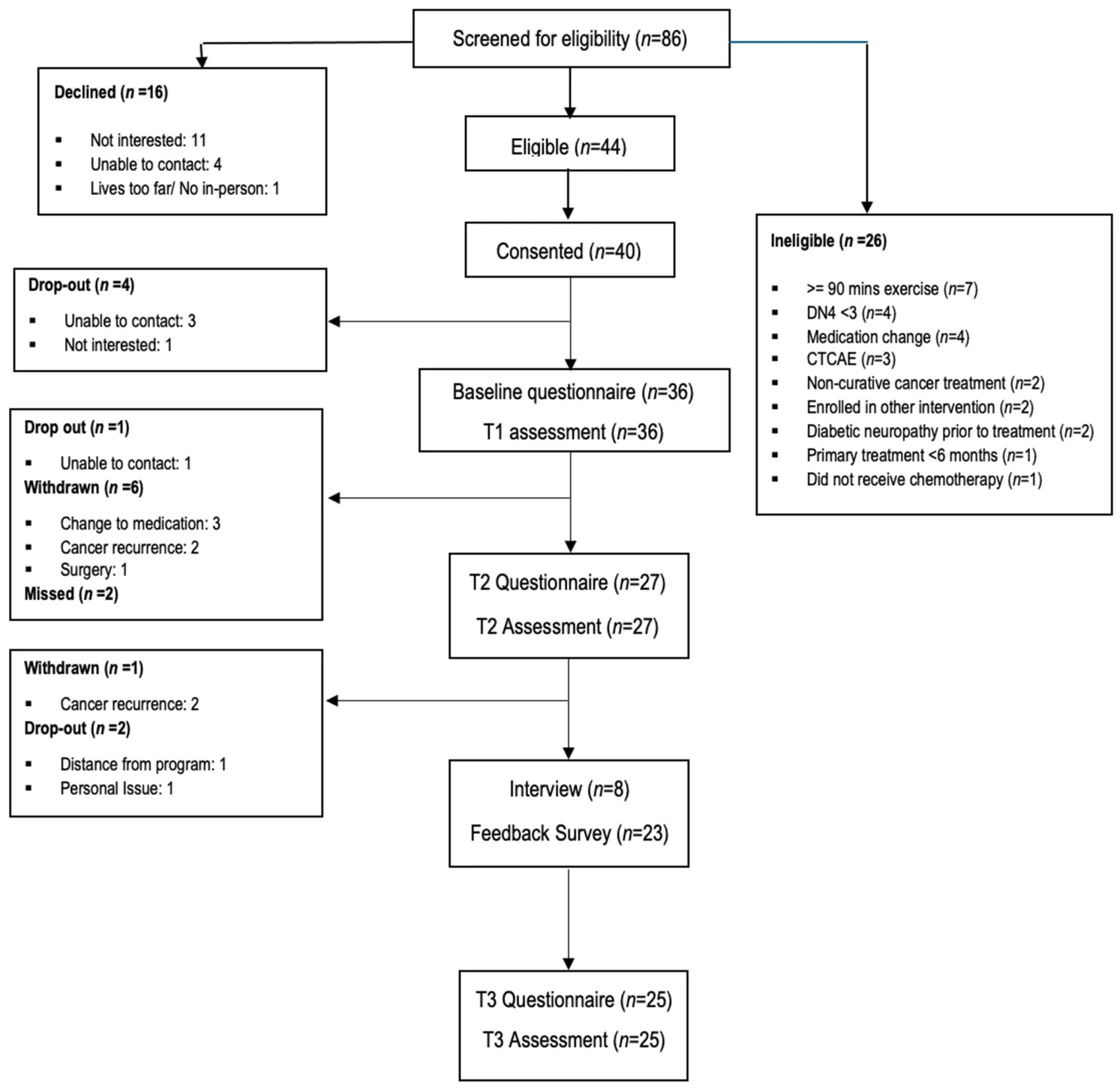
Adapted CONSORT participant flow diagram. Abbreviations: *n*, sample size; DN4, Neuropathic Pain 4; CTCAE, Common Terminology Criteria for Adverse Events.

Study accrual and reasons for withdrawal or dropout are presented in the CONSORT [42] participant flow diagram shown in Figure 1. The rate of consent for eligible participants was 91% and the accrual rate for the study was 3.6 participants per month. The retention rate was 90% at T1, 68% at T2, and 63% at T3. Adherence rate to intervention components was as follows: Check-in calls (96%), reaching weekly aerobic exercise goals (77%), completing weekly resistance and flexibility exercise (85%).

### 3.3. Primary Outcomes—Acceptability

#### Post-Program Feedback Survey

**Figure 2:**
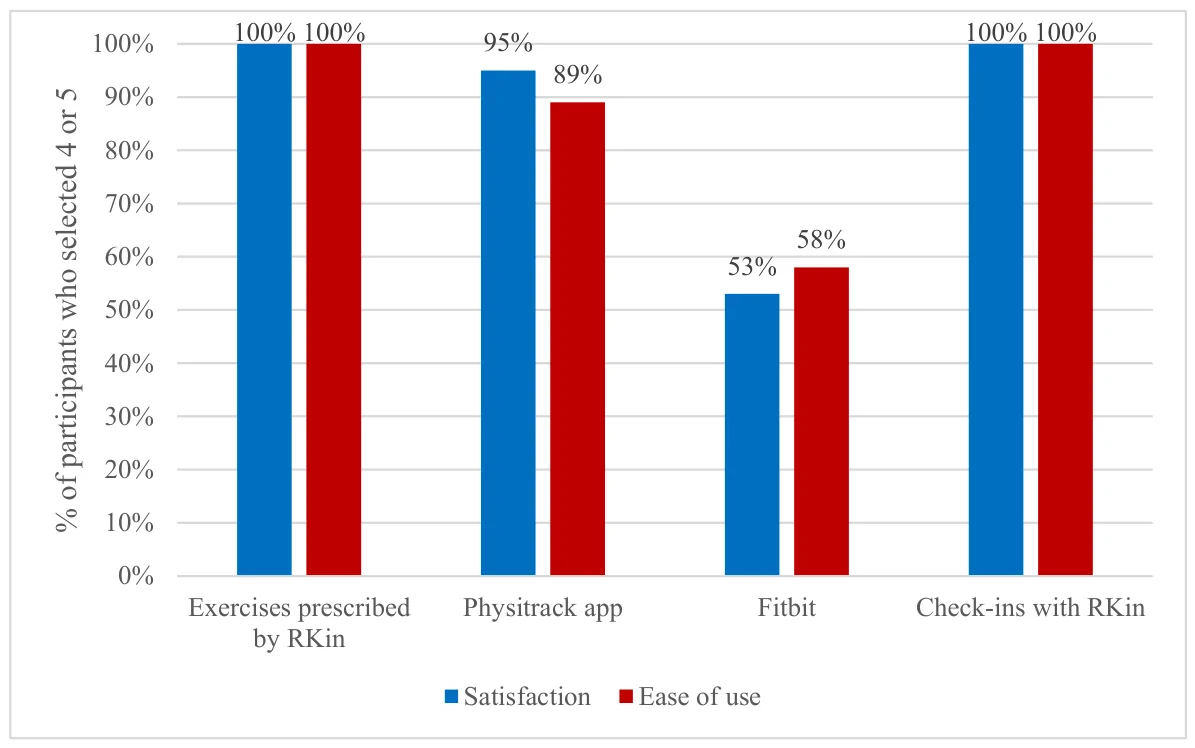
Feedback survey—proportion of participants who selected 4 or 5. Abbreviations: RKin, Registered Kinesiologist.

Twenty-three post-program surveys were completed. A score of 4 or higher out of 5 on the 5-point Likert scale was deemed to demonstrate the participant finding a question acceptable. Acceptability of the program was supported with all participants reporting the usefulness of the program and 95% of participants rating both their satisfaction with the program and the screening and registration process as acceptable. Acceptability of the RKin exercise programs was demonstrated by 100% of participants rating both the satisfaction and ease of use of prescribed exercises and check-in calls as acceptable. For the Physitrack^®^ application, acceptability was shown in 91% of participants for satisfaction and 83% for ease of use. However, 55% of participants rated satisfaction with the Fitbit^TM^ as acceptable with 15% giving a neutral rating, 30% providing a 1 or 2 out of 5, and 4 participants declining to answer as they did not use the device. Similarly, for ease of use of the Fitbit, acceptability was shown in 61% with 17% providing a neutral score, 22% providing a 1 or 2 out of 5, and 4 declining to answer. These data are presented in Figure 2.

### 3.4. Qualitative Interviews

Of 14 approached, 8 individual semi-structured interviews were completed. Interview participants were aged 44–72, the majority had either ovarian cancer (*n* = 4, 50%), or breast cancer (*n* = 3, 38%) as well as colon cancer (*n* = 1, 13%) and had different ethnic backgrounds [Caucasian (*n* = 4, 50%), Chinese (*n* = 3, 38%), and Filipino (*n* = 1, 13%)]. The four themes that arose from the analysis are summarised below with detailed description and individual quotes in Table 3. The full qualitative interview codebook can be found in Supplementary Table S1.

#### 3.4.1. Theme 1: Benefits Experienced by Patients from the EX-CIPN Program

All eight participants expressed obtaining support from the program through study staff and health coaching and touched on general satisfaction with the program supporting quantitative acceptability findings. As a result, all participants emphasised acquiring new knowledge and skills from the program to manage CIPN-specific and more general symptoms. These skills included desensitisation exercises, stretches for feet and calves, balance training, and being more aware of CIPN symptom changes. In total, six participants spoke about experiencing improvement in their CIPN symptoms and overall health and all participants discussed an increased level of motivation and encouragement that helped them focus on further improvement. Improvements of CIPN symptoms included increased sensation, decreased pain, improved toe mobility, and improved balance and coordination. Patients also touched on improvements in muscle strength, muscle tightness, and mental health.

#### 3.4.2. Theme 2: Facilitators of the EX-CIPN Program Benefits

Participants discussed study resources and program design as the main facilitators for program benefits. All interviewees found access to the remote exercise application (Physitrack^®^) to be helpful and five participants found the wearable device (Fitbit^TM^) supportive in completing the program. Participants stated that the visual aspect of Physitrack^®^ application allowed them to focus on technique and the application itself provided motivation by allowing them to track their exercise completion. Those who found the Fitbit^TM^ helpful mentioned that it pushed them to set goals for step count and aerobic exercise minutes which kept them accountable. There were seven participants that expressed satisfaction with the check-in calls and the personalised, progressive nature of the program. They also discussed the acceptability of the program’s remote nature as it made the program easier for them to complete. This was because it helped them to overcome barriers such as travel to in-person sessions and fixed timing of exercises classes that they cannot always attend. All participants found study assessments to be safe, appropriate, and a reasonable length.

#### 3.4.3. Theme 3: Identified Drawbacks of the EX-CIPN Program

Participants felt that there was a lack of long-term follow-up and brought up the want for more in-person assessment and check-in calls over time. There were three participants that described that they experienced a lack of symptom improvement compared to what they expected from the program. This caused some discouragement during the program which led to lowered motivation.

#### 3.4.4. Theme 4: Barriers to Program Attendance and Participation Faced by EX-CIPN Participants

A total of five participants who already had a remote monitoring device reported that they did not like using the study Fitbit as they preferred their own device. Difficulty with transportation to assessments and symptom burden were also reported as barriers to program attendance and completion of the intervention.

### 3.5. Primary Outcomes—Safety

No serious adverse events (AE), defined as grade 3 or higher, related to the intervention or study procedures were observed. Nine grade 1 program-related AEs were documented, all resolving and none resulting in a need to alter study procedures (Table 4). All unrelated AEs can be found in Supplementary Table S2.

### 3.6. Secondary Clinical Outcomes

**Figure 3:**
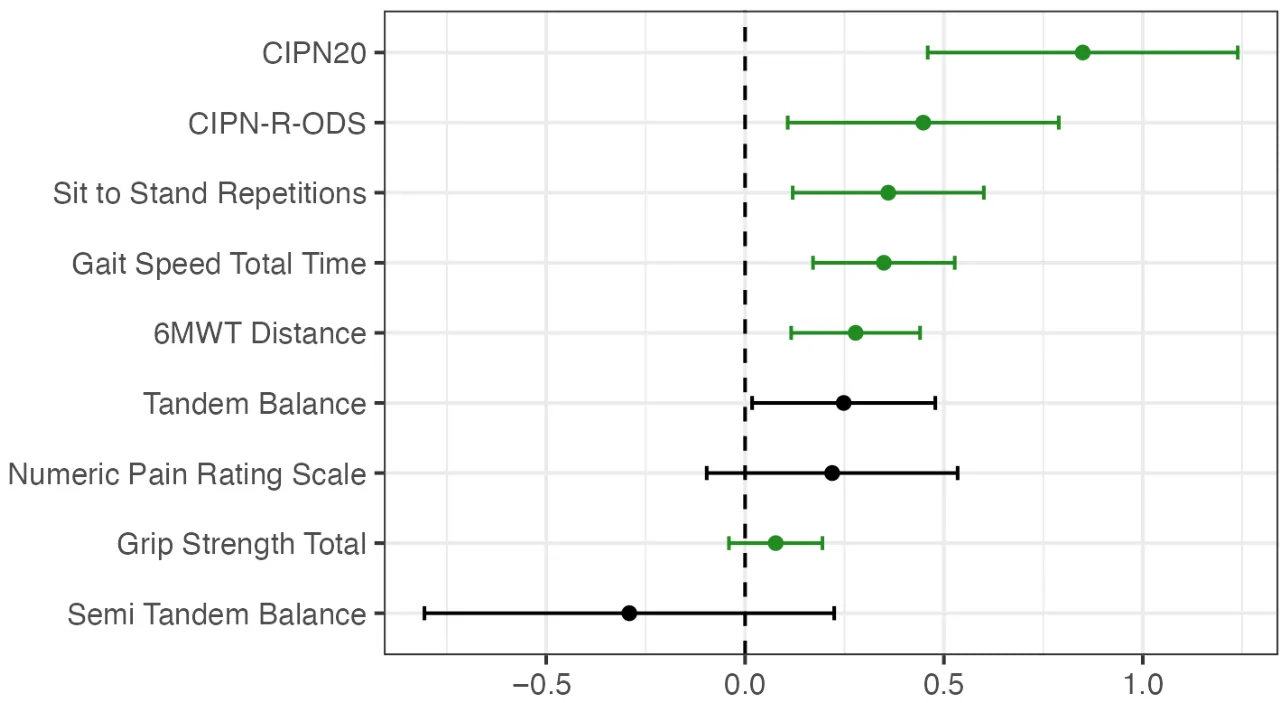
Standardised effect size change from T1 to T2. The black dotted line represents an effect size of 0. Green lines indicate outcomes where the change was statistically significant at T3. Outcomes are ordered by effect size. Abbreviations: 6MWT, six-minute walk test; CIPN-R-ODS, Rasch-built Overall Disability Scale for patients with chemotherapy-induced peripheral neuropathy; CIPN20, European Organisation for Research and Treatment of Cancer Quality of Life Questionnaire—Chemotherapy-Induced Peripheral Neuropathy 20-item scale.

**Figure 4:**
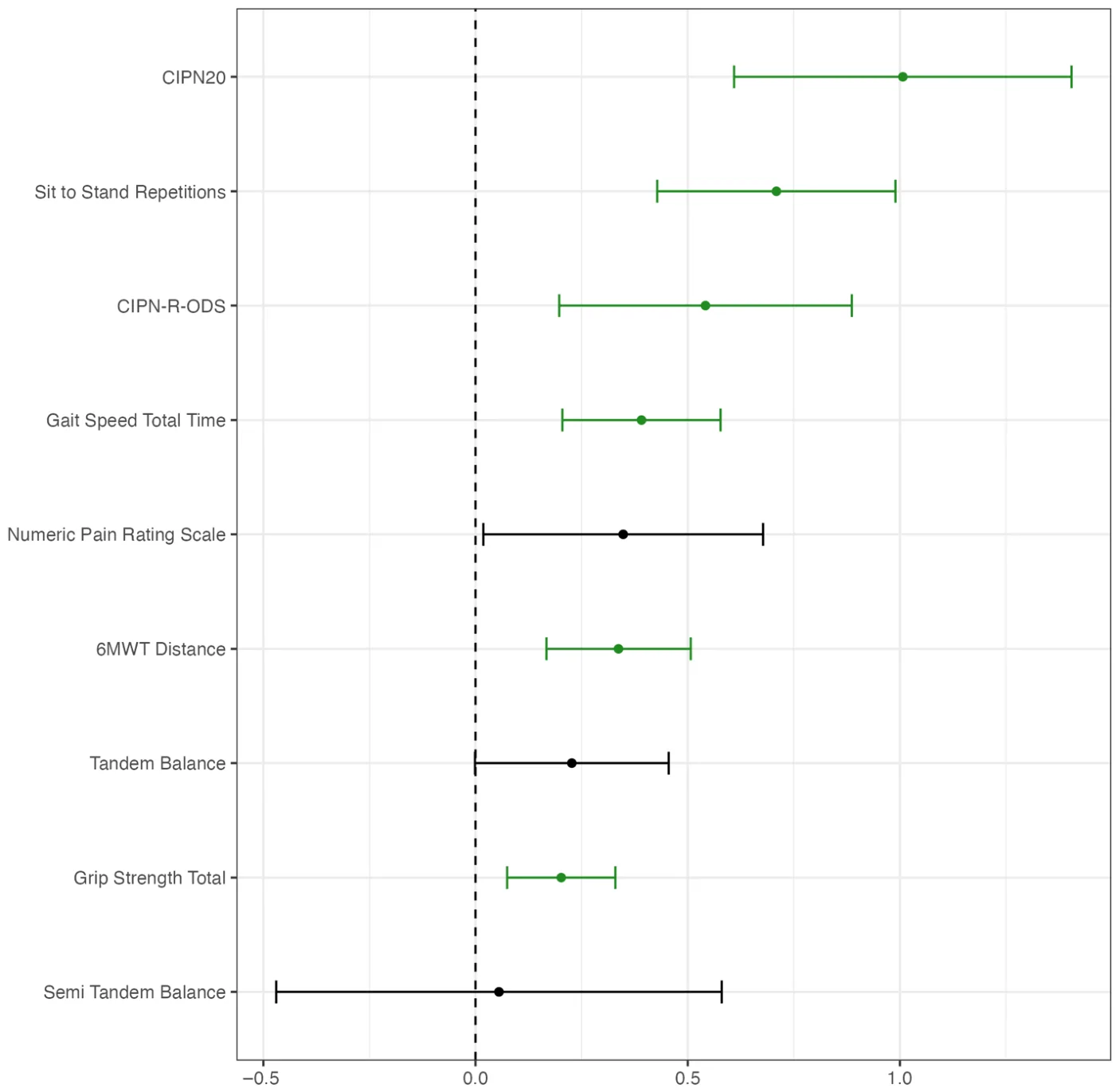
Standardised Effect Size Change from T1 to T3. The black dotted line represents an effect size of 0. Green lines indicate outcomes where the change was statistically significant. Outcomes are ordered by effect size. Abbreviations: 6MWT, six-minute walk test; CIPN-R-ODS, Rasch-built Overall Disability Scale for patients with chemotherapy-induced peripheral neuropathy; CIPN20, European Organisation for Research and Treatment of Cancer Quality of Life Questionnaire—Chemotherapy-Induced Peripheral Neuropathy 20-item scale.

To allow for comparisons across the outcomes, we computed the standardised effect sizes for each of the outcomes. The effect sizes (ES) and 95% confidence intervals (CI) for secondary outcomes from T1 to T2 are shown in Figure 3 and from T1 to T3 are shown in Figure 4. From T1 to T2, a large effect was seen on the EORTC CIPN-20 (ES, −0.85; 95% CI, −1.24, −0.46) and a small-to-moderate effect was seen on the CIPN-R-ODS (ES, −0.45; 95% CI, −0.79, −0.11), gait speed (ES, −0.35; 95% CI, −0.53, −0.17), sit-to-stand (ES, 0.36; 95% CI, 0.12, 0.60), 6MWT (ES, 0.28; 95% CI, 0.12, 0.44), and NPRS (ES, −0.22; 95% CI, −0.53, 0.10).

At T3, there was a large effect for the EORTC CIPN-20 (ES, −1.01; 95% CI, −1.40, −0.61; *p* < 0.001), a moderate effect for the CIPN-R-ODS (ES, −0.54; 95% CI, −0.89, −0.20; *p* = 0.004) and sit-to-stand (ES, 0.71; 95% CI, 0.43, 0.99; *p* < 0.001), and a small effect for gait speed (ES, −0.39; 95% CI, −0.58 to −0.20; *p* < 0.001), 6MWT (ES, 0.34; 95% CI, 0.17, 0.51; *p* < 0.001), grip strength (ES, 0.20; 95% CI, 0.07, 0.33; *p* = 0.001), tandem balance (ES, 0.23; 95% CI, −1.3 × 10^−3^, 0.46; *p* = 0.052), and NPRS (ES, −0.35; 95% CI, −0.68, −0.02; *p* = 0.052). At T3 the patients exceeded the MCID for EORTC CIPN-20 which is defined as a change of 5.06 to 7.32 [61]. MCID was exceeded for sit-to-stand with the MCID defined as a change of ≥2 [62]. MCIDs were also reached for the 6MWT, defined as 14.0–30.5 m [63], and gait speed, defined as 0.08 and 0.11 m/s [53]. Table 5 reports all mean scores and effect sizes at each timepoint.

## 4. Discussion

Overall, the EX-CIPN program and study evaluations were acceptable and safe. We saw encouraging feasibility outcomes overall but missed our set target for follow-up retention rates. Consent of eligible participants was high (91%) and adherence to check-in calls, aerobic, and resistance and flexibility exercise, 96%, 77%, and 85%, respectively, exceeded our feasibility target and were high compared to previous literature with exercise in CIPN [18]. The retention rate at T1 was 90%, reaching the feasibility target of 70%; however, this target was narrowly missed for retention at T2 and T3, 68 and 63%, respectively. Of the 15 participants who dropped out or were withdrawn, 8 were unrelated to the study intervention itself [cancer recurrence (4), physician prescribed changes to medication (3), and surgery (1)]. This speaks to the unpredictability of the population and should be factored into any future sample size estimation. These cancer and CIPN-related factors may also lead to a higher than previously recommended recruitment target for future studies. Safety of the intervention and study procedures was demonstrated through rigorous collection of safety data, no serious AEs, and only mild AEs attributable to the intervention which resolved over time.

Participants found the program duration, frequency of check-in calls, and the remote, individualised style of the program acceptable. This high acceptability is similar to other remote exercise-based interventions in cancer survivorship populations [64,65]. Participants reported that the assessments and questionnaires were easy to complete and showed high levels of satisfaction and ease of use with components of the program including the Phystitrack^®^ app, the exercises provided by the RKin, and the check-in calls. Participants suggested that their engagement would be facilitated by increased follow-up/check in calls in the post-program period. Mixed results were seen with the Fitbit^TM^ device with some participants finding it helpful and some reporting that they did not like using it. The majority of participants who commented negatively about the study Fitbit^TM^ device reported that they already had a wearable tracking device, so they did not enjoy using the study device because they could no longer use their own. Some participants who reported this also found the Fitbit^TM^ helpful regardless, reporting that it kept them accountable with aerobic exercise. As the negative comments surrounding the Fitbit^TM^ were not surrounding a lack of usefulness and the positive comments reported improved motivation and accountability, the Fitbit is still important to include. In the future, we would offer an intervention specific device only to those participants who do not already have a wearable device.

This study adds to the existing literature as it provides patient feedback and preliminary clinical results for an exercise-based rehabilitation program in a persistent CIPN population. To date, most of the literature on exercise-based interventions in CIPN focus on patients post- or during chemotherapy. Further, no studies have been performed on virtual exercise programming in persistent CIPN, so this trial provides novel insights on the feasibility and acceptability of remote exercise in this population [17]. Finally, it is the first study to incorporate behaviour change theory and techniques in a CIPN population and provides data on how this can impact program feasibility and outcomes [27].

### Clinical Outcomes

The preliminary findings on CIPN symptoms, CIPN-related disability, gait speed, grip strength, sit-to-stand, and 6MWT were promising. At T3, the participants were able to reach established MCIDs for the CIPN-20, sit-to-stand, gait speed, and 6MWT. A larger, adequately powered study with a control group is required to validate these findings. The results from this pilot trial will provide estimates and inform sample size calculations to help power a larger randomized trial.

The two outcomes that showed the least improvement over time were the NPRS and SPPB balance. The NPRS had a mean baseline score of 3.20 demonstrating mild pain, and leaving little room for improvement [66]. In CIPN, sensory symptoms such as tingling and numbness are reported most frequently, whereas neuropathic pain symptoms are less commonly observed and when patients are asked to report pain, they do not perceive these sensory symptoms as pain [67]. Moving forward, an alternate primary clinical outcome may be more accurate in the CIPN population (ex. EORTC CIPN-20). Similarly, we found high baseline scores for the SPPB balance test leading to ceiling effects. More sensitive balance assessment, such as the Berg Balance Test, may better capture changes in balance within this population.

## 5. Study Limitations

The EX-CIPN study design has several acknowledged limitations. External generalisability of the study may be constrained, as the study population was highly educated and of higher socioeconomic status, which may not reflect the broader CIPN population. Future research should incorporate purposeful sampling strategies to ensure inclusion of participants across diverse socioeconomic and educational backgrounds. Due to the absence of a control group and a small sample size, the secondary clinical outcomes could not be powered, and findings should, therefore, be considered preliminary. Further, the lack of control group means that changes in outcome data may still be due to natural progression over time as opposed to the intervention itself. Subsequent studies should include larger samples and appropriate control groups to more rigorously evaluate these endpoints. CIPN assessment in this study was based solely on patient reported measures. No electrodiagnostic testing was performed in this study such as nerve conduction studies. This is a limitation as we are able to understand how the intervention affects patient’s neuropathy symptom experience but not how the intervention might affect underlying biologic outcomes such as nerve action potential and nerve growth. These outcomes should be included in future research to understand how exercise interventions can affect underlying biology. Patients with pre-existing neuropathy were excluded to isolate the effects of the program on CIPN symptoms specifically. However, this criterion may have excluded individuals who could potentially benefit from the intervention, such as those with baseline neuropathy that worsened following chemotherapy. The inclusion of patients actively receiving treatments for CIPN, despite allowing at least 6 weeks for treatment results to stabilize, may be a limitation as we cannot be sure that effects of treatment will not surface after the 6-week period. All participants who completed qualitative interviews had reached the T2 timepoint at the time of interview, and none withdrew afterward. This may introduce representation bias, as perspectives from individuals who discontinued participation were not captured. Additionally, the interview sampling was based on convenience, resulting in underrepresentation of certain demographic groups, including males, ethnic groups such as Black and South Asian participants, and cancer diagnoses such as genitourinary cancers. Future studies should adopt a purposeful sampling approach to ensure broader and more diverse representation. Finally, certain potentially confounding variables were not measured, including duration of chemotherapy exposure and length of peripheral neuropathy symptoms, which may have influenced outcomes.

## 6. Conclusions

The EX-CIPN pilot study provides valuable insights into the feasibility, acceptability, and safety of a remote exercise-based rehabilitation program in persistent CIPN. Results from this study can help to refine future program design and present important information on implementing rehabilitation in CIPN populations. Despite promising preliminary clinical outcome findings, larger scale studies using randomized designs are required to determine the effectiveness of the EX-CIPN program.

Based on our findings from this study, we plan to adjust our intervention and study design for future research. The main adaptations that we plan to make to the intervention include the addition of a 6-month post-program follow-up assessment and a supported maintenance phase with two monthly health coaching calls between T2 and T3. This will address the participants want for more check-in calls and in-person assessments over time while also helping to assess the longer-term impacts of the EX-CIPN program to investigate the continued outcome improvement that we saw at T3. For future research, we also plan to introduce exploratory subgroup analyses by gender, sex, type of chemotherapy, time since chemotherapy completion, dose of chemotherapy, and baseline symptom level. This will help us to gain insight into which patients benefit most from the intervention and for whom the treatment may need modification.

## Figures and Tables

**Table 1:** Behaviour change techniques used in EX-CIPN.

Behaviour Change Technique Group	EX-CIPN Program Components	Behaviour Change Techniques Present
Goals and Planning	Goal discussion at weekly check-in calls with RKin Goal review at follow-up assessments with RKin	Goal setting (behaviour), Problem-solving, Goal setting (outcome), Action planning, Review behaviour goal, and Discrepancy between current behaviour and goal.
Monitoring	Physitrack^®^ self-monitoring FitBit^TM^ self-monitoring	Self-monitoring of behaviour, Self-monitoring of outcome(s) of behaviour.
Social Support	Support provided by RKin at assessments One-on-one support provided during weekly check-ins by RKin	Social support (unspecified), Social support (practical), and Social support (emotional).
Repetition and Substitution	Repetitive weekly exercise prescriptions Exercise progression monitored by RKin	Behavioural practice/rehearsal, Behavioural substitution, Habit formation, Habit reversal, Generalisation of a target behaviour, and Graded tasks.
Self-belief	RKins provide support, reassurance, and guidance	Verbal persuasion about capability and Self-talk.

Abbreviations: RKin, Registered Kinesiologist.

**Table 2:** Baseline participant demographics and medical characteristics.

Variable	Total Participants (*n* = 36)
Age (years)	
Mean (SD)	60.3 (10.9)
Median (range)	59.0 (39–77)
Diagnosis *n* (%)	
Breast	16 (44)
Ovarian	11 (31)
Colon	7 (19)
Testicular	1 (3)
Uterine	1 (3)
Cancer stage *n* (%)	
Stage II	13 (36)
Stage III	13 (36)
Stage I	7 (19)
Stage IV	3 (8)
Type of Chemotherapy *n* (%)	
Taxane and platinum-based	12 (33)
Platinum-based	12 (33)
Taxane	8 (22)
Other	4 (11)
Gender *n* (%)	
Female	30 (83)
Male	6 (17)
Ethnicity *n* (%)	
White/Caucasian	25 (69)
Chinese	5 (14)
Black	3 (8)
South Asian	1 (3)
Filipino	1 (3)
Other	1 (3)
Education *n* (%)	
Completed college/university	19 (53)
Post-graduate degree	7 (19)
Professional degree	5 (14)
Completed high school	4 (11)
Other	1 (3)
Employment *n* (%)	
Retired	17 (47)
Full time	8 (22)
Not currently employed	3 (8)
Long-term disability	3 (8)
Part time	1 (3)
Self-employed—Full time	1 (3)
Self-employed—Part-time	1 (3)
Temporarily on sick leave	1 (3)
Other	1 (3)
Socioeconomic status *n* (%)	
>$75,000	16 (44)
Prefer not to answer	8 (22)
$20,000-$39,000	7 (19)
$40,000-$75,000	4 (11)
<$20,000	1 (3)
Living arrangement *n* (%)	
Lives with spouse/partner	23 (64)
Lives alone	10 (28)
Living with other family/friend	3 (8)
CIPN medication use at baseline *n* (%)	
Not taking medication	28 (78)
Taking medication	8 (22)
Pregabalin	6 (75)
Duloxetine	2 (25)
CIPN complementary therapy use at baseline *n* (%)	
Not using complementary therapy	34 (94)
Using complementary therapy	2 (6)
Acupuncture	2 (100)
NPRS (units)	
Mean (SD)	3.2 (2.6)

Abbreviations: *n*, sample size; SD, standard deviation; NPRS, numeric pain rating scale; CIPN, chemotherapy-induced peripheral neuropathy.

**Table 3:** Qualitative interview results.

Theme	Subtheme	Definition	Example
Benefits experienced by patients from the EX-CIPN program	New knowledge and skills acquired to manage general and CIPN-specific symptoms	Participants found that the EX-CIPN program provided them with strategies that were helpful in CIPN and general symptom recovery.	“I still definitely, I will roll the ball, and then even one of the suggestions … put my feet in a bucket of rice, you know to try to bring, you know, to stimulate, I do that. But not all of them.”
	Experienced improvement in CIPN symptoms and overall health	Through the consistent exercise program and learned strategies, participants found improvement in their CIPN and overall health.	“Before my fingers, when I use my fingers to touch the touch screen on the cellphone. It’s often no response. Now it’s still sometimes no response, but not as frequent as before.” “…like after do those exercise, the hands and the feet feel warmer. Like feel better.”
	Obtained support from the program	Participants emphasised the support from study staff through check-in calls, appointment flexibility, and expertise in designing the exercise programs.	“Because ___ [study staff—kinesiologist] explained very detail. I know like the program it’s designed by the specialist, so I believe you were helping me. That’s why I continue the program.” “___ [study staff—kinesiologist] did very good it’s easy to communicate yeah.”
	EX-CIPN increased motivation and encouragement to improve health behaviours	Participants felt that the program provided them with extra motivation and encouragement to exercise consistently by developing routine and providing accountability.	“I think because, usually I stay, I just sit in the office to work and not very active to do the exercises. Now it’s important to like to start to even move the body (laughs) move the hands so it’s a habit to be more active.”
Facilitators of the EX-CIPN program benefits	Provided access to helpful resources	Participants found the use of the Fitbit to be helpful for motivation and expressed that the Physitrack application helped them to complete exercises from home.	“I think having the videos was a good way to do it because people are visual. And you want to be able to see if you are doing it right. Because I lot of those stretches you don’t get it right until, a couple days after and it’s like “oh now I can see” “now I can feel that little nerve floss stretch” “now I understand what they’re saying”.”
	Program design was effective	Participants spoke about how they enjoyed the personalised, progressive design of the exercise program. They found the program was not too time consuming and they were satisfied overall.	“I’ll be honest when we heard how many appointments there was gonna be, we were like “ugh I can’t commit to that”. Then it was like they’re all video calls and it was like “that I can commit to”
	No concerns with the study requirements	Participants had no issues with the questionnaires or physical assessments and found them to be acceptable.	“There was nothing overtly, strenuous you know? I think it was all very doable. And I think the length wasn’t too bad. We were there like 35 40 min or so. And ____ [study staff—kinesiologist] was on time which was nice”
Identified drawbacks of the EX-CIPN program	Program delivery could be improved	Participants had suggestions on program delivery including less repetitive exercise programming and more study visits and check-in calls.	“But I’m kind of like bored because I’ve been doing the same thing again and again right.”
	Lack of symptom improvement was found	Some participants expressed a lack of symptom improvement compared to what they expected. This led to some lost motivation.	“Like for me, not having any positive change in my neuropathy I don’t think anything would be, you know (pauses) moving forward likely stand to continue to do it on a daily weekly monthly basis type thing.”
Barriers to program attendance and participation faced by EX-CIPN participants	Difficulty with transportation to assessments	Some participants reported transportation to downtown Toronto as a barrier due to traffic and commute time.	“You know the biggest, I guess sort of pain, was coming down right to the hospital.”
	Symptom burden affected participation	Symptom burden related to cancer diagnosis or treatment affected some participants’ ability to complete certain exercises.	“Like some days my shoulder is hurting so I don’t do certain things.”
	Underutilisation of program tools	When provided with a Fitbit, some participants expressed that they did not use it as they already had their own device.	“My only reluctancy honestly was, you know changing out a FitBit for my watch.”
	Felt a lack of recruitment affected study participation	Some participants felt that it took a long time to find out about the study and that advertisement or recruitment should be increased to help with recruitment closer to 6 months post-chemotherapy.	“…the only thing I can suggest is that it should be advertised like a little bit more because like I had gone to all of my regular check ins like every four weeks at the clinic, and you know the nurses there, the PAs, the oncologist, everyone knew about my symptoms, but it wasn’t until I saw a new PA, because I guess the other one was not in that day or whatever.”

Abbreviations: CIPN, chemotherapy-induced peripheral neuropathy; PA, physician’s assistant.

**Table 4:** Program-related adverse events reported by EX-CIPN participants.

Adverse Event	CTCAE Category	Grade	# of Participants
Leg/back muscle soreness	Myalgia	1	4
Foot/ankle/heel discomfort	Pain in extremity	1	3
Calf cramping at night	Muscle cramp	1	1
General Fatigue	Fatigue	1	1

Abbreviations: CTCAE, Common Terminology Criteria for Adverse Events.

**Table 5:** Outcome mean scores and effect sizes at different time-points.

Outcome	Time-Point	Mean Score	Score Change from T1 (95% CI)	ES (95% CI)	*p* Value
NPRS	T1	3.17			
	T2	2.64	−0.52 (−1.3, 0.2)	−0.22 (−0.53, 0.10)	
	T3	2.33	−0.83 (−1.6, −0.1)	−0.35 (−0.68, −0.02)	0.052
**EORTC CIPN-20**	T1	32.89			
	T2	21.86	−11.03 (−15.7, −6.3)	−0.85 (−1.24, −0.46)	
	T3	19.81	−13.08 (−17.8, −8.4)	−1.01 (−1.40, −0.61)	**<0.001**
**CIPN-R-ODS**	T1	73.43			
	T2	58.22	−15.22 (−26.4, −4.1)	−0.45 (−0.79, −0.11)	
	T3	55.03	−18.41 (−29.6, −7.3)	−0.54 (−0.89, −0.20)	**<0.001**
Semi tandem balance	T1	9.97			
	T2	9.84	−0.13 (−0.4, 0.1)	−0.29 (−0.81, 0.22)	
	T3	10.00	0.03 (−0.2, 0.3)	0.06 (−0.47, 0.58)	0.83
Tandem balance	T1	8.38			
	T2	9.07	0.69 (0.1, 1.3)	0.25 (0.02, 0.48)	
	T3	9.02	0.63 (0.0, 1.2)	0.23 (−1.3 × 10^−3^, 0.46)	0.052
**Grip strength**	T1	50.53			
	T2	52.13	1.60 (−0.8, 4.0)	0.08 (−0.04, 0.20)	
	T3	54.76	4.23 (1.9, 6.6)	0.20 (0.07, 0.33)	**0.001**
**4MGS**	T1	4.02			
	T2	3.59	−0.44 (−0.6, −0.2)	−0.35 (−0.53, −0.17)	
	T3	3.54	−0.49 (−0.7, −0.3)	−0.39 (−0.58, −0.20)	**<0.001**
**Sit-to-stand**	T1	9.83			
	T2	11.70	1.87 (0.7, 3.0)	0.36 (0.012, 0.60)	
	T3	13.51	3.67 (2.5, 4.8)	0.71 (0.43, 0.99)	**<0.001**
**6MWT**	T1	443.72			
	T2	479.91	36.18 (17.6, 54.8)	0.28 (0.12, 0.44)	
	T3	487.69	43.97 (25.1, 62.8)	0.34 (0.17, 0.51)	**<0.001**

Bolded outcomes represent outcomes that reached statistical significance at T3. Abbreviations: 6MWT, six-minute walk test; 4MGS, 4 metre gait speed; CIPN-R-ODS, Rasch-built Overall Disability Scale for patients with chemotherapy-induced peripheral neuropathy; EORTC CIPN-20, European Organisation for Research and Treatment of Cancer Quality of Life Questionnaire—Chemotherapy-Induced Peripheral Neuropathy 20-item scale; NPRS, Numeric Pain Rating Scale.

## Data Availability

The original contributions presented in this study are included in the article/Supplementary Materials. Further inquiries can be directed to the corresponding author.

## Supplementary Materials

The following supporting information can be downloaded at: https://www.mdpi.com/article/10.3390/curroncol33100621/s1, Table S1: Qualitative Interview Codebook; Table S2: Unrelated Adverse Events reported by EX-CIPN Participants.
