# Supplementary material for: A Remotely Delivered Exercise-Based Rehabilitation Program for Patients with Persistent Chemotherapy-Induced Peripheral Neuropathy (EX-CIPN): A Phase I Feasibility Trial
Source: Curr Oncol. 2026 Oct 15;33(10):621. doi: 10.3390/curroncol33100621 (PMC13703083; doi:10.3390/curroncol33100621)
Supplement: Supplementary file 1 [file curroncol-33-00621-s001.zip › Table S1.pdf]

Supplementary Table S1. Qualitative Interview Codebook

Theme	Subtheme	Code	Code definition	Example
Benefits experienced by patients from the EX-CIPN program	New knowledge and skills acquired to manage general and CIPN-specific symptoms	Applied skill/strategy acquired from EX-CIPN	The participant applied and is continuing to apply a skill or strategy learned in the EX-CIPN program. This can include their exercise plan, a new exercise or way of exercising, walking, planning their schedule etc. The participant discusses how they implemented something they learned in the program.	"I still definitely, I will roll the ball, and then even one of the suggestions, it wasn't a part of the program, but you know maybe, out my feet in a bucket of rice, you know to try to bring, you know, to stimulate, I do that. But not all of them."
		Learned new skills/strategies in EX-CIPN	This code refers to discussion on how participants were taught a new skill/piece of knowledge or a variety of skills. This way the participant could do something they may not have done without the program or could choose which exercises worked best for them. Includes discussion of the breadth of the program, the information taught, the variety of exercises etc.	"I'm just learning the different exercises that I wouldn't have and then doing those, okay great I've learnt something new that I can use to try to get rid of this."

	Experienced improvement in CIPN symptoms and overall health	Improved neuropathy or physical symptom	This code refers to discussion on how the program directly improved a neuropathy specific or a general physical symptom exerienced by the participant. The participant may discuss the direct effect of the prescribed exercises or the effect of the support from the RKins. Improvment may include physical functioning, fitness, pain, tingling, numbness, sensation, fatigue, etc.	"Before my fingers, when I use my fingers to touch the touch screen on the cellphone. It's often no response. Now it's still sometimes no response, but not as frequent as before." "...like after do those exercise, the hands and the feet feel warmer. Like feel better."
		Improved psychological/mental health	This code refers to discussion on how the program had a positive impact on the psychological, psychosocial, or mental health of the participant. The participant may have found an improvement in mental or psychological health due to exercise or RKin support during the program.	"Yes, mental health yes"

		Improved overall health	This code refers to discussion on how the program had a positive impact on the participants overall health. Generally broad statements on how the program impacted the health of the participant. The participant does not specify what was improved, or whether it was physical or psychological, just that the program had a positive impact on their health and helped them feel better.	“Yeah, and so that was great cause I really- I was really happy to see that resting heart rate start to settle again.”
	Obtained support from the program	Appreciated flexible appointment scheduling	This code refers to discussion on the scheduling of the program and its flexibility. This includes discussion on the ease of booking in-person assessments, health coaching calls or any other communication throughout the study.	"It was pretty good, I mean he (study staff - kinesiologist) was pretty flexible too, like I would try to do it, when I had other appointments downtown already, so I wasn't coming down just to see him (study staff - kinesiologist)."
		Appreciated the support from program staff	This code refers to discussion on the support of the EX-CIPN staff. This includes discussion	"Because ___ [study staff – kinesiologist] explained very detail. I know like the program

			on support from the RKins and other staff. This can include mention on the expertise and knowledge of the staff, the encouragement, the assistance and advice from staff, adaptations made by staff, and the kindness and understanding from staff.	it's designed by the specialist, so I believe you were helping me. That's why I continue the program." " ___ [study staff – kinesiologist] did very good it's easy to communicate yeah."
		Presented general satisfaction with the EX-CIPN program	The participant discussed that they were generally satisfied with the program or that they found general benefit or support from the program.	"In general I think it was pretty good."
	EX-CIPN increased motivation and encouragement to improve health behaviours	Helped to create a routine	The participant found that the EX-CIPN program helped them create a new routine and gave them structure. The EX-CIPN program helped them start and maintain an exercise routine, helped them get up in the morning, or gave them skills that they implement on a day-to-day basis.	"I think because, usually I stay, I just sit in the office to work and not very active to do the exercises. Now it's important to like to start to even move the body (laughs) move the hands so it's a habit to be more active."

		Provided the potential for neuropathy improvement	The participant found that the potential for neuropathy improvement or any improvements made to their neuropathy symptoms during the program kept them motivated to complete the program exercises and/or calls during and after the program completion.	"well maybe, hearing that, it could possibly help. You know I don't think I would've been given the exercise I was given if it wasn't potentially going to help so I kind of just hoped that it would."
		Provided accountability for exercise behaviours	This code refers to discussion on how the program held the participant accountable to their goals and routine. Perhaps participants felt they needed to show up for their instructors, or they wanted to see improvement in their neuropathy and fitness, or perhaps they have seen some improvements and these improvements have motivated them to be accountable for themselves.	"And it does remind me, I need to be more active if I want to (pauses) to get better like."

Facilitators of the EX-CIPN program benefits	Provided access to helpful resources	Found the use of the FitBit to be helpful	This code refers to the use of the Fitbit as a support during the EX-CIPN program. The participant found the FitBit to be helpful in supporting their exercise journey.	"Yeah I use it [FitBit]..."
		Access to Physitrack supported completion of exercise	This code refers to discussion on the Physitrack application and the use of the application while completing the EX-CIPN program. The participant may discuss the frequency of use, the benefits of use, and what they found helpful about this application. The participant may mention how Physitrack helped them remember their exercises and provided them guidance and instructions on how to do them. The participant may mention how it was helpful the exercises could be adjusted in Physitrack as they progressed and changed with the support of the	"I think having the videos was a good way to do it because people are visual. And you want to be able to see if you are doing it right. Because I lot of those stretches you don't get it right until, a couple days after and it's like "oh now I can see" "now I can feel that little nerve floss stretch" "now I understand what they're saying"."

			kinesiologist. They may have used Physitrack to track their exercise completion and to hold them accountable.	
	Program design was effective	Found check-in calls to be useful in their exercise journey	This code refers to discussion on the check-in calls with the RKins, weeks 2, 3, 4, 6, and 8 of the program. This discussion can be on the benefit of the calls, the ease of the online calls, the content discussed during the calls, or the frequency/duration of the calls. The participant may have found that the calls held them accountable, gave them support, and helped them with their exercise journey.	"I'll be honest when we heard how many appointments there was gonna be, we were like "ugh I can't commit to that". Then it was like they're all video calls and it was like "that I can commit to"
		Appreciated the personalized exercise program	This code refers to discussion on the benefit of receiving a personalized exercise plan from the RKin based on their own specific needs and goals. This can include discussion on how the participant appreciated	"So (pause) being very flexible with that was very good and not just being like "here's the program" "everyone has to do the same exercise, we all have to do the same or there's no

			receiving and individualized plan, how this helped them start exercising or learning how to exercise properly, how they felt the plan supported their goals, how exercise progression was specific to them, or how they felt safer knowing this was specific to them.	point in the study" it wasn't like a study."
		Preferred the individual-based style of program	This code refers discussion on participants' preference for an individual program as opposed to a group setting. This can include discussion on how the participant felt more comfortable completing the program individually, if it helped them to start exercising, if it helped them in deciding to take part in the program, etc.	I – "Yeah, yeah. So, correct me if I'm wrong but did it appeal to you that this was an individual program then?"<br><br>P – "Yeah, yeah. 100%."
		Appreciated the progressive design of the exercise program	This code refers to discussion on the benefit of having progression with exercises throughout the program. This	"I kind of increased them but I guess __ [study staff - kinesiologist] did give me extra, extra exercises you know

			can be discussion on the progression of exercises provided by the RKin, the autonomy to progress certain exercises on their own and how this helped them to remain accountable and supported their goals.	every time we checked in, if it was you know going well and stuff like that. So that was beneficial."
		Liked the remote format of the program	Participants found that the remote format was helpful, valuable, and acceptable. Participants may have enjoyed the ease of the remote program delivery, felt it erased barriers to attending in-person programs, or felt it helped them to remain more frequent in their exercise as they could complete it from home.	"For me I think it was good because I'm from out of town. I'm coming in from _____. Only needed to actually be face to face in the hospital, you know, two or three times was very easy for us to be able to do. And just have a video call or a check in whatever it was, every two weeks or so, was very feasible to be able to do at home."
		Felt that the exercises were not a time burden	Participants shared that they appreciated that the exercises did not take too long to do. This led to increased consistency.	"because the exercises, even with what I was doing it couldn't have been more than twenty minutes a day. For me to

			This suggests that if they took a long time it would be harder to be consistent.	squeeze that in was very easy … Like I could always find the time.”
		Satisfied with the overall program duration	This code refers to discussion on the length of the 10-week program. This discussion can be on the total amount of weeks in the program and how this length benefitted the participants and their exercise journey.	"You know, i think it was 12, but whatever, yeah it was, based on when we scheduled the appointments, but no I think it's a good, you know, time period because usually, if you're doing something regularly for three month period and you find, or feel like it works well, you're more likely to continue doing it."
	No concerns with the study requirements	Found the physical assessments to be acceptable	The participant had no issues with the assessments. They did not feel like the assessments were too long, invasive, or unsafe.	"there was nothing overtly, strenuous you know? I think it was all very doable. And I think the length wasn’t too bad. We were there like 35 40 minutes or so. And ____ [study staff – kinesiologist] was on time which was nice"
		Found the questionnaires to be acceptable	The participant had no issues with the questionnaire package.	"Yeah I just did that. I don’t remember the one I did in the

			They did not feel like the questionnaires were too long and they felt the questions asked were appropriate and applicable.	beginning but I'm sure I did it. The one I just did, super easy."
Identified drawbacks of the EX-CIPN program	Program delivery could be improved	Found the exercise program to be repetitive	This code refers to discussion on how the exercise program can be repetitive. The participant discusses how they would like some parts of the program to differ or change therefore maintaining engagement in the program.	"But I'm kind of like bored because I've been doing the same thing again and again right."
		Suggested an increase in in-person study visits	The participant would have liked more in-person visits or times to reset of the exercise program mid-way through the program to try new exercises if no changes in symptoms were found.	"I don't know maybe, like if halfway through there wasn't like any sort of improvements, there could be any type of changes or adaptations to the exercises."
		Found the exercises felt too easy	Discussion on how the participants felt certain aspects or exercises within the program felt easy. They may have felt	"I think (pause) for me a lot of the activities and exercises felt a little, let's say easy or remedial."

			that neuropathy exercises did not challenge them physically.	
	Lack of symptom improvement was found	Experienced no improvement in neuropathy symptoms	Discussion on how the participant did not feel any changes in their neuropathy symptoms. They may have felt that the exercise program was helpful in other aspects of their life and overall strength, but were discouraged by the lack of improvement in neuropathy.	"Like for me, not having any positive change in my neuropathy I don't think anything would be, you know (pauses) moving forward likely stand to continue to do it on a daily weekly monthly basis type thing."
Barriers to program attendance and participation faced by EX-CIPN participants	Difficulty with transportation to assessments	Long distance to the hospital affected ease of assessments	This code refers to discussion on how patients may have found it difficult to commute to the hospital. This couls include discussion on distance to the hospital, traffic, transit problems, etc.	"You know the biggest, I guess sort of pain, was coming down right to the hospital."
	Symptom burden affected participation	Physical symptoms affected participation in the program	This code refers to how the participant was experiencing physical symptoms related to cancer and its treatment and this posed a barrier to participation in the EX-CIPN program. May	"Like some days my shoulder is hurting so I don't do certain things."

			have been a barrier to physical exercise or travelling to the program.	
	Underutilization of program tools	Did not like using the FitBit	The participant did not use the FitBit possibly because they didn't like wearing it, they weren't interested in using it, it was uncomfortable, they were confused on how to use it, or they forgot to make use of it.	"Also sometime I feel a little bit itchy maybe my skin too sensitive or maybe it's skin is not so good after chemo. So sometimes I put it on and I don't feel comfortable, I take it off and put it somewhere where I don't wanna lost it so I (laughs). "
		Preferred to use a personal device	The participant did not enjoy using or did not want to use the Fitbit as they had their own personal device that they wanted to keep using and felt that they liked more.	"My only reluctancy honestly was, you know changing out a FitBit for my watch."
	Felt a lack of recruitment affected study participation	Suggested an increase in study recruitment	The participant feels it took a long time for her to find out about the study and that further clinics or areas of UHN and outside hospitals could be informed of the program	"...the only thing I can suggest is that it should be advertised like a little bit more because like I had gone to all of my regular check ins like every four weeks at the clinic, and

			through further advertisement, so that the program can be heard about earlier in treatment.	you know the nurses there, the PAs, the oncologist, everyone knew about my symptoms, but it wasn't until I saw a new PA, because i guess the other one was not in that day or whatever."
