# Supplementary material for: A Remotely Delivered Exercise-Based Rehabilitation Program for Patients with Persistent Chemotherapy-Induced Peripheral Neuropathy (EX-CIPN): A Phase I Feasibility Trial
Source: Curr Oncol. 2026 Oct 15;33(10):621. doi: 10.3390/curroncol33100621 (PMC13703083; doi:10.3390/curroncol33100621)
Supplement: Supplementary file 1 [file curroncol-33-00621-s001.zip › Table S2.pdf]

Supplementary Table S2. Unrelated Adverse Events reported by EX-CIPN Participants

Ongoing shoulder discomfort	Pain	Grade 1 – Mild, did not limit instrumental ADLs	Unrelated – Regular shoulder tightness and discomfort from previous surgery, not increased by exercises
Ankle pain	Pain in extremity	Grade 2 – Moderate, limited some instrumental ADLs	Unrelated – The patient reported ankle pain due to rolling their ankle while walking down the stairs
Left foot swelling	Edema limbs	Grade 1 – Mild, swelling noticeable only on close inspection	Unrelated – Regular swelling in the foot from previous treatments, not increased by the program
Swelling in right arm	Edema limbs	Grade 2 – Moderate, readily apparent deviation from normal anatomic contour	Unrelated – Reported fluid buildup in the right arm due to previous cancer treatments
Fall	Fall	Grade 1 – Minor with no resultant injuries	Unrelated – Reported tripping on a pipe while taking the garbage bin out to the street
Fall	Fall	Grade 1 – Minor with no resultant injuries	Unrelated – Reported tripping while walking up the stairs and falling forwards
Ongoing shoulder pain	Pain	Grade 1 – Mild, did not limit instrumental ADLs	Unrelated – Regular shoulder pain from previous surgery, not increased by exercises

Abbreviations: ADL, activities of daily living
